# The impact of a reduced fertility rate on women's health

**DOI:** 10.1186/1472-6874-4-S1-S11

**Published:** 2004-08-25

**Authors:** Jennifer Payne

**Affiliations:** 1 Centre for Chronic Disease Prevention and Control, Health Canada, 120 Colonnade Rd, Ottawa, Canada

## Abstract

**Health Issue:**

Total fertility rates (TFRs) have decreased worldwide. The Canadian fertility rate has gone from 3.90 per woman in 1960 to 1.49 in 2000. However, not many studies have examined the impact on women's health of reduced fertility rates, delayed fertility and more births to unmarried women. This paper presents information on the relation between family size and specific determinants of health.

**Key Findings:**

The rate of TFR decline varies considerably by geographic location and socio-demographic subgroup. Further, the associations between family size and selected determinants of health are different for women and men. For example a woman with one child is almost four times more likely to be "coupled" than a childless woman, and if she has two children she is significantly more likely to be "coupled" than if she had only one child. However, a man with one or more children is over six times more likely to be "coupled" than his childless counterpart, and this does not vary with family size.

**Data Gaps and Recommendations:**

There is a paucity of data on the impact of reduced fertility rates on women's health in general and on how women's roles affect their decision to have children. While it would be useful to examine longer-term health outcomes by parity and age of first birth, as well as socio-economic and role-related variables these longitudinal and detailed "role related" data are not available. Given the differing profiles of women and men with children, further health policies research is needed to support vulnerable women with children.

## Background

**Figure 1:**
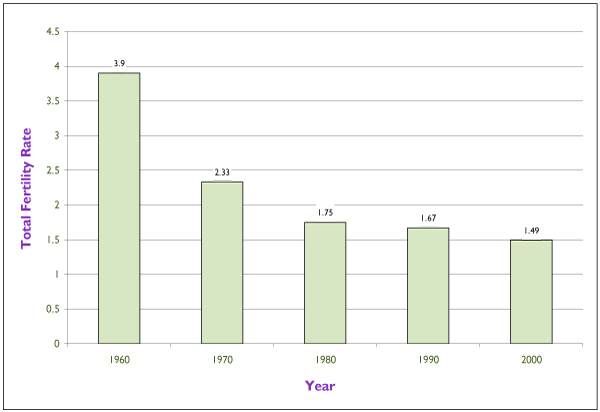
**Total Fertility Rate for Canada 1960–2000 **Source: Statistics Canada, Vital Statistics, 1960–2000

Over the last four decades, total fertility rates (TFRs, or number of children each woman bears on average) have decreased worldwide, and particularly in developed countries such as Canada (Figure 1). The Canadian fertility rate decreased by over 60%, from 3.90 per woman in 1960 to 1.49 in 2000, below the replacement level of 2.1 children per woman. [1,2] However, the question of how these changes in TFR have affected women's health has not been studied extensively in the literature. [3]

This chapter presents information on fertility trends in Canada and worldwide (between 1960 and 2000), and some of the associations between parity and health in the literature. As well, it examines the pattern of family size by various socio-demographic variables, and the association between family size (number of children) and the likelihood of specific health-related outcomes (such as having a partner, having less than high school education, being unemployed, and perceiving one's health to be "good to excellent") in women and men. The factors associated with the intention to have children were also examined.

### Trends in Fertility Rate

#### Worldwide Trends

Between 1960 and 2000, TFR decreased for all regions, although the decrease was most pronounced for the less developed regions, including all regions of Africa, Latin America and the Caribbean, Asia (excluding Japan), Melanesia, Micronesia and Polynesia. [4] Many factors have contributed to the reduction in TFR, including increases in women's level of education, [5] access to effective contraception, delay in marriage, and culture. [6,7] Although national fertility surveys in the United States and Canada in 1995 suggested that Canadian and American women want to have the same number of children – on average 2.2 – the 2000 TFR in Canada was 1.49, [6] as compared with 2.06 in the United States, [8] a gap that has increased by two thirds in the last 20 years. An analysis of the gap shows that 60% of the difference is due to the different fertility rates among women aged 20 to 29 years, and an additional one third is due to the gap in teen fertility (15 to 19 years). The age-specific fertility rate among women 30 years and older is the same in the two countries. [6] Hypotheses suggested for the difference in TFR between the two countries include (i) the difference in time of marriage (marriage occurs earlier and more often in the United States), (ii) difference in time of first childbirth, which occurs, on average, at 29 years in Canada versus 27 years in the United States, (iii) easier access to contraceptives in Canada, and (iv) a stronger economy and lower unemployment in the United States. [6]

#### Provincial and Territorial Trends

**Figure 2:**
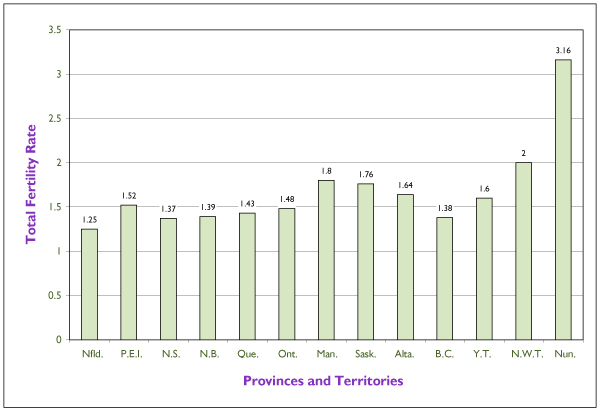
**Provincial/territorial Total Fertility Rate 2000 **Source: Statistics Canada, Vital Statistics

Within Canada, the TFR varies considerably by province and territory. The lowest fertility rates are in the Atlantic provinces (Newfoundland and Labrador, New Brunswick, and Nova Scotia) and in Quebec and British Columbia. The highest levels are in the Prairie provinces (Manitoba, Saskatchewan and Alberta) and all of the Territories. These higher fertility rates are believed to be due, in part, to the greater Aboriginal population (with more younger women and higher fertility) in these provinces and territories (Figure 2). In 2000, the number of live births fell in all provinces and territories in Canada except the Northwest Territories, where it rose 2.1%. [2]

### Social and Economic Trends

#### Mother's Age

During the period of 1969 to 1999, the mean age of mothers increased from 23.7 years to nearly 29 years, and between 1986 and 1999 a steadily increasing proportion of births occurred among women 30 years and older (from 29% in 1986 to 45% in 1999). [9,10] However, fertility has decreased overall, and the largest reduction in age-specific fertility in Canada in the last 30 years has occurred among women between 20 and 24 years of age and among teens, by 59% and 55% respectively. The rate in the 25 to 29 age group also decreased, by about 33%. On the other hand, the fertility rate among women between 30 and 34 has stayed the same or decreased at a slower rate. [9-11] Similarly, the proportion of first births in the 20 to 24 age group and the 15 to 19 age group declined (from 46% to 24%, and 24% to 11%, respectively) between 1971 and 1999, whereas among women over 25 it increased in all age groups. [10]

#### Marriage/Partnership

A decrease or at least a delay in marriage, especially in developed countries, has contributed to the decline in fertility in spite of an increase in non-marital child-bearing. [12-15] In Canada in the 1950s, only about 4% of births were to unmarried mothers, [9] by 1990 it had increased to 24% [16], and by 1999 to 32%. [10] Marriage, while still important, seems to have become less of an issue for women as they receive more education and participate more in the workforce. [17] Dupuis, analyzing the General Social Survey (GSS) 1995, found that Canadians who were married or planned to marry intended to have more children than those who were not planning to get married. [18] Dupuis also showed that the popularity of common-law relationships is increasing in all age groups but that such relationships are most frequent among younger people. These data are consistent with the statistics that show an increase in the proportion of births to unmarried women in almost all provinces and territories. [9,10] This trend is particularly evident in Quebec and the Northwest Territories, where the proportion of births to unmarried women increased by 25% and 15% respectively between 1986 and 1996. [11]

#### Education

Many researchers [19-22] have documented the association between education, particularly educational attainment of the mother, and fertility. However, some research has suggested that the trend towards postponed child-bearing has occurred primarily among women with at least high school education [3,23] and, further, that educational level-specific fertility rates may not be declining. [5]

#### Ethnicity

Culture is an important factor in determining the number of children a woman will have in her lifetime. U.S. data show large differences between the various ethnic groups. In the United States in 2001, the TFR was lowest for non-Hispanic whites, at 1.85 children per woman, and highest for Hispanics (3.17). [25] Similarly in Canada, the fertility rate among Aboriginal Canadians is one and a half times that among non-Aboriginal Canadians, [24] and there are also differences in fertility rates between Canadian-born women and foreign-born Canadians. [25] Nizalova, studying the social and economic impact of maternity leave duration in 160 countries, found that the effect of similar maternity policies varied by country grouping. Longer maternity leaves resulted in higher fertility rates in certain countries and lower rates in others. These researchers suggested that local culture and traditions were an important factor in determining the rates. [26]

#### Economic Well-Being

The link between poverty and TFR has been well documented in the literature and is illustrated by the association between TFRs and gross national income (GNI per capita adjusted for purchasing power). [27] However, there is evidence that in developed countries an increase in uncertainty about future income may lead to postponement of child-bearing. [28] Haub suggests that a lack of confidence in the economy and insufficient income were important factors in the Russian decline in fertility, from 1.9 in 1990 to 1.4 in 1993. [29] Similarly, higher economic uncertainty in Canada was among the reasons given for the growing gap in TFR between the United States and Canada over the last 20 years. While unemployment rates were similar for young Canadians and young Americans in the early 1980s, they were consistently higher among young Canadians in the 1990s. [6]

#### Decreasing Parity and Disease Risk

While it seems obvious that having children can affect the quality of an individual's life socially and psychologically, it is less generally recognized that having children and having a particular number of children affects a woman's pattern of disease risk. Research has shown associations between parity and several diseases. Examples include breast, ovarian, endometrial, uterine, colorectal, cervical and renal cancers, rheumatoid arthritis, leukemia, diabetes, Alzheimer's disease, urinary incontinence and obesity. Both high parity and a young age at first pregnancy have been associated in the literature with a reduction in breast cancer risk, [30-34] although recent research suggests that this reduction in risk may differ with age and ethnicity. Palmer et al. found that among African-American women younger than 45 years, parity was associated with an increased risk of breast cancer and among women 45 years and older was associated with a decreased risk. [35] Similarly, having children (as well as incomplete pregnancies) has been shown to be protective against ovarian cancer, particularly for those with a family history of ovarian cancer. [36-38] Several research groups have also shown that high parity and late age at first birth are protective against endometrial cancer. [39-43] Associations between parity and reduction in risk of osteoporosis, [44,45] rheumatoid arthritis, [46] some leukemias, [47] colorectal cancer [48] and diabetes [49,50] have also been reported. On the other hand, high parity has been associated with an increased risk of cancer of the cervix, among women testing positive for human papillomavirus, [51] Alzheimer's disease, [52] urinary incontinence, [53-55] obesity, [56] cholesterol gallstones [57] and renal cell cancer. [58] High parity has also been associated with risk factors for heart disease, such as the increase of carotid artery plaques, lower levels of HDL (high-density lipoprotein cholesterol) and high glucose-insulin ratios long after child-bearing has ceased. [59]

It is clear that a better understanding of the changing pattern of women's disease risks with parity would be useful to women making decisions about their fertility as well as to health practitioners developing women's health-related policies and programs. More work is needed in this area.

## Methods

### Data Sources

Data from the GSS Cycle 10, The Family, 1995, were used to look at the distribution of family size by socio-demographic characteristics, the association of family size with specific determinants of health, and also factors associated with the intention of having children in the future. The GSS 1995 focused on family and marital history (marriage and common-law relationships), family origins, brothers and sisters, children, fertility intentions, marriages, values and attitudes towards certain areas of family life, paid and unpaid work, and health perception. It included individuals aged 15 and over and excluded full-time residents of institutions and residents of the Territories. These data were weighted to represent the Canadian population. For the multivariate analysis rescaled weights were used. This technique takes into account the unequal probabilities of selection, but does not take into account the stratification and clustering of the sample's design. [60]

### Variables

The variables used were as follows:

#### Family Size

This was based on the number of children born to females or males 15 years or older. It was divided into five categories: none, 1, 2, 3 or 4, and = 5 children.

#### Age

This was grouped into three categories: 15–29, 30–49, and = 50 years.

#### Marital Status

Categorized as single never married individuals, married (including common-law and married couples), and previously married (separated, divorced or widowed individuals).

#### Educational Level

Categorized as "less than high school" and "high school or more."

#### Income

Household income was used, divided into two categories: "less than $40,000" and "$40,000+." Approximately 30% of the records were missing for this variable. The data were not available to examine income adequacy, which takes into account family size.

#### Home Ownership

This referred to whether someone in the household owned the accommodation and was categorized as "own yes or no." This was used as a proxy for income, as the two variables were somewhat correlated. The correlation coefficient between these variables was 0.33, which in these data was significant, p < 0.0001.

#### Employment Status

Categorized as "no employment in the last 12 months" or "some employment in the last 12 months."

#### Ethnicity

This variable was grouped as "North American," "European" and "Other." Unfortunately, the samples for other ethnic groups were too small to analyze.

#### Region

Categorized as Atlantic (Newfoundland and Labrador, Nova Scotia, P.E.I. and New Brunswick), Quebec, Ontario, Prairies (Manitoba, Saskatchewan and Alberta) and British Columbia.

Self-perceived health: Divided into "Excellent, Very good or Good" versus "Fair or Poor."

To study the association between family size and selected determinants of health (presented in Figure 4), "family size" was chosen as the independent variable and modelled with different outcome variables. The five outcomes considered were (i) marital status re-categorized as a bi-variate variable single (including never married as well as previously married) or couple (married or common-law), (ii) educational level, (iii) employment status, (iv) home ownership and (v) perceived health. In each case the model included family size and was adjusted for age, ethnicity and the four outcome variables that were not the outcome variable in the particular model.

Four value statements from the GSS were also included in the analysis in Figure 5, which looked at the factors associated with the intention to have children in the future. They were (i) having a child is important to happiness in life, (ii) what a women really wants is a home and children, (iii) a woman should refuse a promotion at work if it means spending too little time with her family, and (iv) a man should refuse a promotion at work if it means spending too little time with his family. For the first statement the respondents had to rate the extent to which having a child was important versus not important. Important was used as the comparison category. For the other three statements the respondents were asked to indicate the extent to which they agreed with the statements. Two agreement categories were collapsed and two disagreement categories were collapsed. The collapsed agreement category was used as the comparison.

## Results

### Distribution of Family Size by Socio-demographic Characteristics (Figures 3a to 3c)

These data show that a greater proportion of women have children than men and that they have them younger. Women 50 years and older were almost twice as likely to have had a large family of 5 children or more as men aged 50 and older. (However, one needs to bear in mind that the men were still biologically able even though the proportions did not change appreciably after 50 years.) As would be expected, single women and men were less likely to have had children than those who were married or previously married. However, single women were about twice as likely as men to have had one or more children.

Women with more than high school education were almost 20% more likely to be childless than those who had less than a high school education, whereas those with less than high school were at least four times as likely to have a large family (≥ 5 children) than those with more than high school. For men the difference was in the same direction but less dramatic. Income also seemed to be negatively associated with family size. However, as discussed earlier, the quality of this variable is questionable, as many respondents did not answer the question. Employment was negatively associated with having children and, for women, with having large families. Women who had worked in the previous 12 months were almost twice as likely to have no children than those who had not worked in the previous 12 months, whereas women who had not worked were at least six times more likely to have a large family than those who had worked. As with education, the pattern was similar for males but less dramatic.

Analysis by ethnicity suggested that there are differences in fertility patterns by ethnic background, but because of the small numbers and the large area grouping necessary, these data were not useful. There were no discernible regional differences for women or men. However, in Quebec, Ontario and the Prairie regions women were more likely to have children than men. Living in a house owned by a household member appeared to be associated with having one or more children for both women and men. However, it did not affect the likelihood of having a large family. Perception of health was associated with having children and with family size in both women and men: the perception of fair/poor health was more likely in those with one or more children than in those with none.

#### Multivariate Assessment of the Relation Between Number of Children and Selected Determinants of Health (Figure 4)

##### The Relation Between Family Size and Marital Status, Adjusting for Other Potential Confounders

For both women and men the odds of having a partner were significantly higher if the individual had 1 or more children. When compared with women with no children, the odds of a woman with 1, 2, 3–4, and = 5 children having a partner were 3.75 (confidence interval [CI] 3.06, 4.61), 5.90 (CI 4.86, 7.17), 5.33 (CI 4.32, 6.57) and 4.86 (CI 3.67, 6.42) respectively. Further, women with 2 children were significantly as likely to have a partner as those with just 1 child. Men with 1 or more children were more than five times as likely to have a partner as men without children, and this did not vary significantly with the number of children.

##### The Association Between Family Size and Educational Level, Adjusting for Other Potential Confounders

Women with 2, 3–4, and = 5 children were significantly more likely to have less than high school education than women with no children or 1 child, odds ratio (OR) 1.26 (CI 1.01, 1.57), OR 1.83 (CI 1.47, 2.29) and OR 3.50 (CI 2.63, 4.67) respectively. Further, the probability of not having finished high school was significantly higher among women with = 5 children than those with 3 or 4 children. Similarly among men, those with 3–4 or 5 or more children were more likely (OR 1.45, CI 1.14, 1.83 and OR 1.50, CI 1.05, 2.14 respectively) to have a lower education than those with 2 children or less.

##### The Association Between Family Size and Employment Status, Adjusting for Other Potential Confounders

For women, having 1 or more children was directly associated with being unemployed. This ranged from an OR of 1.88 (CI 1.49, 2.36) for a woman with 1 child as compared with a woman with none, to an OR of 3.48 (CI 2.52, 4.82) for a woman with 5 children or more as compared with a woman with none. However, for men, being unemployed was only significantly associated with having 5 children or more, OR 1.81 (CI 1.21, 2.70).

##### The Association Between Family Size and Home Ownership, Adjusting for Other Potential Confounders

The relation between having children and owning a home appeared to be stronger for men than women. Men who had 1, 2, 3–4, or = 5 children were more likely to live in a home owned by one of the occupants than men without children: OR 1.29 (CI 1.04, 1.61), 2.13 (CI 1.72, 2.64), 1.93 (CI 1.52, 2.45) and 1.61 (CI 1.10, 2.36), respectively. The data suggest that those who have 2 children are even more likely to own than those who have 1. Women with 2 children were more likely to own than those with none, OR 2.13 (CI 1.72, 2.64). However, for women, other family sizes were not associated with owning the family home.

##### The Association Between Family Size and Perceived Health, Adjusting for Other Potential Confounders

There was neither a strong nor a consistent relation between number of children and perceived health in women and men. Women who had 2 children were less likely to perceive their health to be excellent, very good or good than those with none. On the other hand, men who had = 5 children were more likely to perceive their health to be excellent, very good or good than those with none.

#### Relation between Values and Age and Educational Level in Women and Men (Figure 5)

Over 70% of both men and women believed that "having at least one child is important to happiness in life." This belief was similar for both sexes, increased with age group, and was more prevalent in those with less than high school education. The proportion varied from 74.96% (CI 71.73%, 78.19%) in younger women, aged 15 to 29 years, to 86.67% (CI 84.97%, 88.37%) in women 50 years or older, and from 76.36% (CI 73.07%, 79.65%) in men aged 15 to 29 years to 84.88% (CI 82.55, 87.21%) in those aged 50 years or older.

Similarly, the belief that "A job is all right, but what most women really want is a home and children" was more common among older individuals and those with lower education; there was no difference between women and men. These proportions varied from 41.04% (CI 37.50%, 44.58%) in younger women, aged 15 to 29 years, to 69.74% (CI 66.73%, 72.75%) in women 50 years or older, and from 46.53% (CI 42.88%, 50.18%) in men aged 15 to 29 years to 73.61% (CI 70.44%, 76.78%) in those aged 50 years or older.

With respect to the belief that "A man should refuse a promotion at work if it means spending too little time with his family," younger men (< 50) were more likely to agree than younger women, whereas older women (= 50 yrs) were more likely to agree than older men. Among women, agreement increased with age, from 41.04% (CI 37.82%, 44.26%) to 69.74% (CI 65.23%, 74.25%). Among men, while those 30 to 49 were more likely to agree with the statement than those 15 to 29 years (58.59%, CI 55.37%, 61.81%, versus 50.36%, CI 47.10%, 53.62%), both of these age groups were similar to those 50 years or older (55.12%, CI 51.55%, 58.69%). There was no difference by educational level for either women or men.

The belief that "A woman should refuse a promotion at work if it means spending too little time with her family" increased with age. The proportion rose from 41.04% (CI 37.82%, 44.26%) of women 15 to 29 years of age to 69.74% (CI 66.73%, 72.75%) of those 50 years or older, and from 52.56% (CI 49.16%, 55.96%) of men aged 15 to 29 years to 68.09% (CI 64.75%, 71.43%) of those 50 years or older. However, in females the belief was more prevalent among those with less than high school education, 67.8% (CI 64.48%, 71.12%), than those with high school education or more, 61.13% (CI 59.33%, 62.93%), whereas in males the prevalence did not vary with educational level.

#### Factors Associated with the Intention of Having One or More Children in the Future (Figure 6)

Multivariate analysis was carried out adjusting for socio-demographic factors, including marital status, education, employment, age (15–19 years or 30–49 years), perceived health, and presence or absence of children. Having more than a high school education most affected the intention to have one or more children in the future among both women and men. However, the pattern was slightly different among women and men. Among women, being employed full time was associated with the intention to have one or more children, whereas among men full-time employment reduced the probability that they intended to have children in the future. Also, while being married did not affect women's intention to have children in the future, it decreased men's likelihood of wanting future children. As one would expect, currently having one or more children or being between 30 and 49 years of age significantly reduced the likelihood of wanting children in the future for both women and men. Women who perceived their health as excellent to good were significantly more likely to intend to have children in the future. Of the four value statements included, only the beliefs that "having a child is important to happiness in life" and that "what a woman really wants is a home and children" were significantly associated with the intention to have children in the future in both women and men.

## Discussion

While birth rates and women's fertility are decreasing in general, worldwide they vary widely between regions and countries. Within Canada our data show large fertility differences among provinces and territories and within subgroups of women. These differences suggest the need for policies and programming targeted to the specific subgroup, perhaps at a provincial/territorial or at a more local level, rather than a global "one size fits all" policy. The increase in average maternal age and average maternal age at first birth could have an impact on maternal mortality and morbidity rates, as has been suggested by some authors. [61,62] Similarly, these women may be more likely to require reproductive and other technologies, which may lead to an increased need for these services. It would be useful to monitor the proportion of births to women 35 years or older as well as to put in place surveillance mechanisms to monitor the demand for reproduction-related technologies.

**Figure 3:**
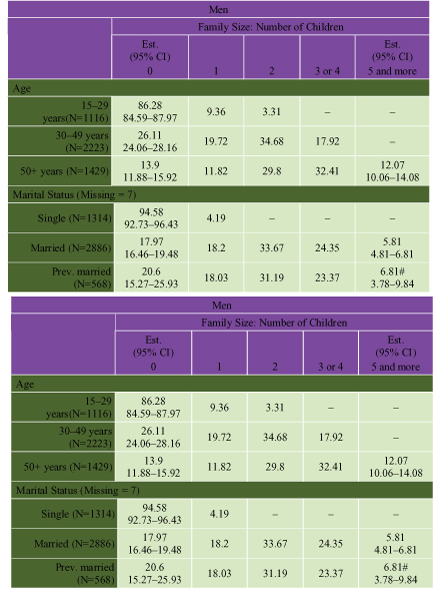
**Showing the Sex Specific Distribution of Family Size by Age and Marital Status **Data Source: GSS Cycle 10, 1995

**Figure 4:**
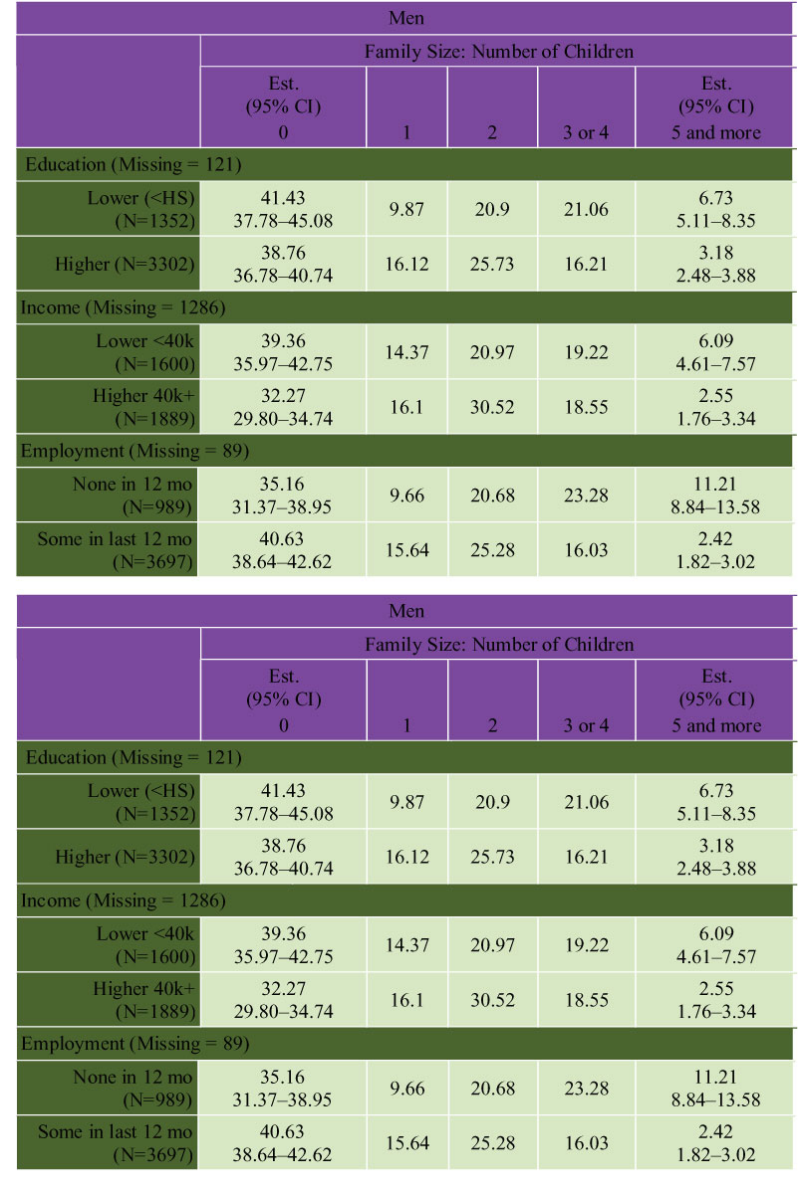
**Showing the Sex Specific Distribution of Family Size by Education, Income and Employment **Data Source: GSS Cycle 10, 1995

**Figure 5:**
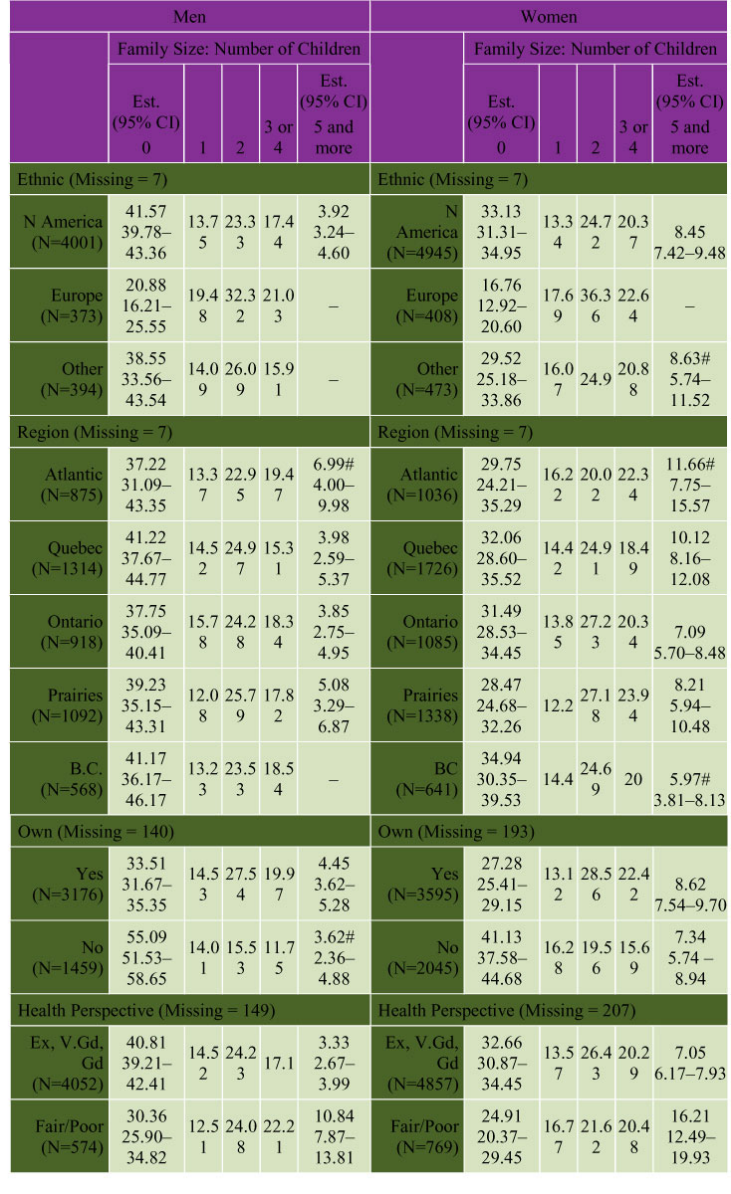
**Showing the Sex Specific Distribution of Family Size by Ethnicity, Region, Home Ownership and Perspective of Health **Data Source: GSS Cycle 10, 1995

In our data, a greater proportion of women had children and had large families (= 5 children) than men. This may, in part, be due to the fact that (i) in this society women tend to form marital or common-law partnerships at a younger age than men [63] and (ii) women aged 50 years or older have finished having their children whereas men aged 50 years or older have not necessarily finished. Subgroups of women less likely to have children and large families were those with more than high school education, higher income and employment; those perceiving their health to be good, very good or excellent; and younger, single women. While some of the associations seen in Figures 3a to 3c are due to the fact that these data are not age-adjusted, association of lower fertility with younger cohorts, being unmarried, having a higher education level, and being employed are consistent with findings reported elsewhere. Factors traditionally associated with reduced fertility, such as the proportion of women not in conjugal unions, the proportion using contraception, the proportion of women not fecund (primarily as a result of breast-feeding), and the level of induced abortions may have contributed to this reduction somewhat but do not seem to be the main force behind the changing fertility patterns in Canada. For example, although the marriage rate in Canada decreased from 8.9 per 1,000 populations in 1971 to 5.1 in 1997, the proportion of births to unmarried mothers tripled between 1986 and 1999.

### Relation Between Family Size and Specific Determinants of Health

Multivariate models looking at the relation between family size and various health-related determinants suggest that the relation is different for women and men. While a woman who has 1 child is about four times as likely to be married as a woman with none, she is less likely to be married than if she had 2 children. On the other hand, a man with a child is about six times as likely to be married as one without children, and this does not seem to vary with number of children. This is consistent with other data suggesting that women are more likely to be bringing up a child without the support of a partner. This can affect health from a psychological as well as an economic perspective. [64,65] Further, it should be noted that, unlike women who have 2 or more children, women who have 1 child are not more likely to have less than high school education nor are they less likely to live in an occupant-owned home than those without children. However, they are more likely to perceive their health as fair or poor. On the other hand, women with 5 children or more are more likely to be of low education and unemployed compared with those with none. Health policies need to take into account that women are more likely than men to be lone parents, and this is likely to have an impact on their ability to work and on their perceived health. Research to find the most effective support mechanisms is needed.

### Factors Associated with the Intention of Having Children in the Future

For women the intention to have a child in the future was directly associated with educational level and employment, in contrast to actual family size, which was inversely associated with educational level and employment. This is consistent with the findings of others [66] that women are choosing higher education and, as a result, are putting off having children.

Perceived excellent to good health, and working, were also associated with the intention to have a child. This makes sense – as women who are in good health and employed may be better able to cope – especially since marital status was not a significant factor. The results of the "values/attitude" variables, the belief that "having a child is important to happiness in life," and the belief that "what a woman really wants is a home and children" were as would be expected. The belief that a woman or man should refuse a promotion at work if it means spending too little time with family was not associated with the intention to have a child among either women or men. It was interesting to note the similarity in responses by males and females for all of the value variables, suggesting that gender roles vis-à-vis family may be less clearly delineated than they once were.

### Data and Knowledge Gaps

The reduction in child-bearing in the last 40 years has clearly contributed to some of the obvious improvements in women's maternal and reproductive health, such as the significant decreases in rates of maternal mortality and other pregnancy complications. While various studies have shown associations between parity and the risk of various health outcomes, there has not been a systematic assessment of the longer-term impact of current fertility patterns, such as reduced fertility rates, delayed fertility, and increased rates of birth to unmarried women, on women's health. Unfortunately, the data were not available to do this analysis. Further, with respect to women's roles, such as marital status, employment and child-care, there was no information about their quality, and this may affect the impact of the changing fertility patterns on women's health.

## Recommendations

• Data on parental and family relationships and data on health are not available in the same databases, making it difficult to examine associations between family status and health risk factors and outcomes. Since women's social roles are believed to have an impact on their healthstatus, data on roles as well as on risk factors and health outcomes should be collected in the same database.

• Further data on the quality and/or conditions of the relationship also need to be provided. Presumably the effects of having a partner, having a job, being a parent, caring for children or adults, and so on depend on the duration and conditions of the relationship, and the individual's perception of the situation.

• Ethnicity and culture appear to be important factors in determining fertility patterns. To examine this further, these factors have to be more clearly defined, and more contextual data need to be provided. Often individuals' experience of ethnic factors is based on the perceptions and reactions of the community as well as on their own.

• The cross-sectional nature of the GSS makes it impossible to capture temporal relations between associated variables. Longitudinal data providing socio-demographic information linked to outcome variables would make it possible to examine the longer-term impacts of the changing fertility rates.

• A greater proportion of women than men have children alone, and women with lower education and no jobs outside of the home seem to have larger families than those with jobs and higher education. Further research is needed to determine the health policies that can best support these most vulnerable women.

## Note

The views expressed in this report do not necessarily represent the views of the Canadian Population Health Initiative, the Canadian Institute for Health Information or Health Canada.

## Figures and Tables

**Figure 6:**
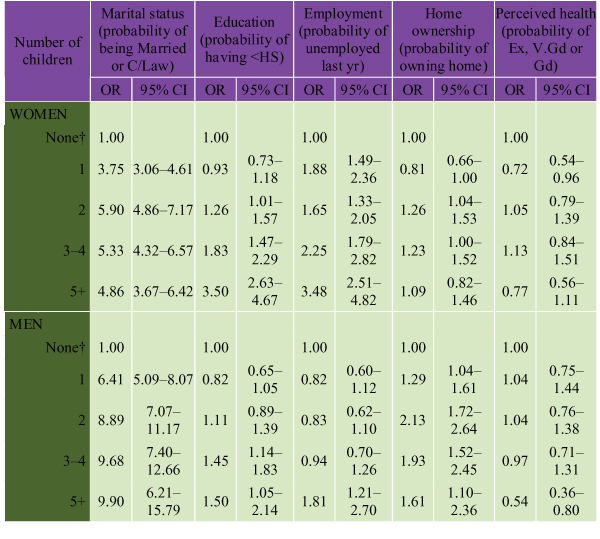
**Multivariate Associations Between Number of Children and Specific Factors Linked to Determinants of Health **Data Source: GSS Cycle 10, 1995

**Figure 7:**
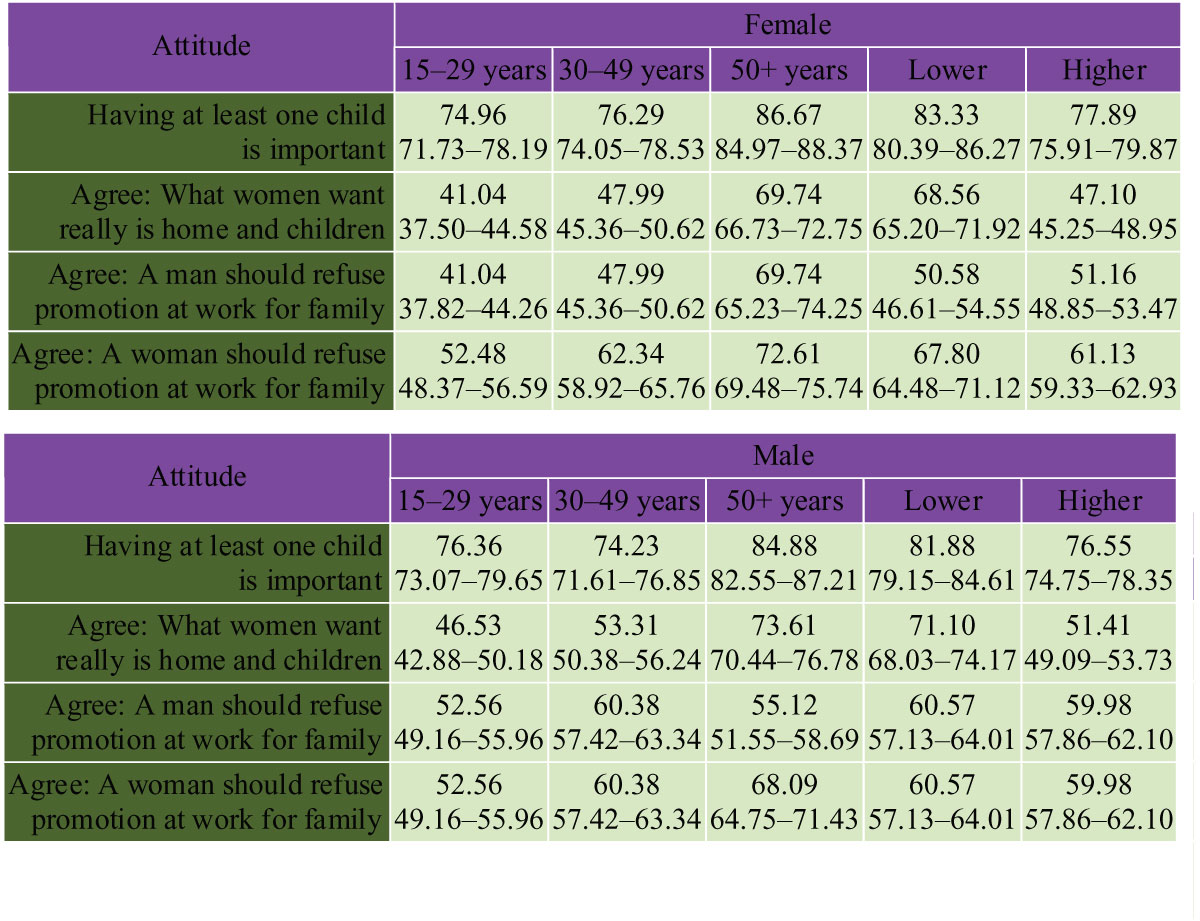
**Distribution of Attitude/Values by Age and Education**. Data Source: GSS Cycle 10, 1995

**Figure 8:**
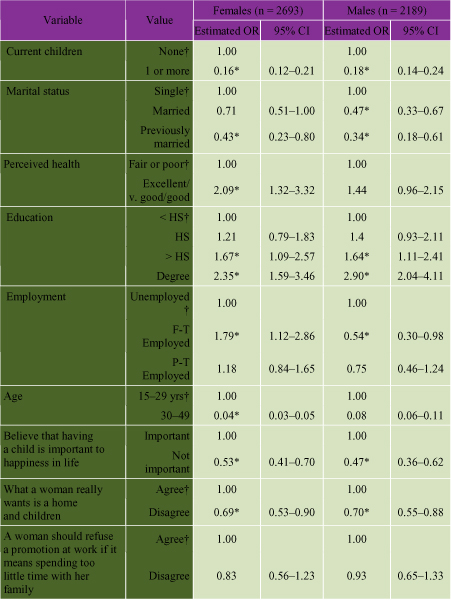
**Results of Multivariate Analysis of Factors Associated with Plans to Have One or More Children in the Future Rescaled Weight Used. **Data Source: GSS Cycle 10, 1995
